# Chronic Pain and Associated Factors Related to Depression among Older Patients in Hanoi, Vietnam

**DOI:** 10.3390/ijerph18179192

**Published:** 2021-08-31

**Authors:** Anh Trung Nguyen, Trang Huyen Thi Nguyen, Thu Thi Hoai Nguyen, Huong Thi Thu Nguyen, Thanh Xuan Nguyen, Tam Ngoc Nguyen, Anh Lan Nguyen, Linh Gia Vu, Hoa Thi Do, Linh Phuong Doan, Giang Thu Vu, Huong Thi Thanh Nguyen, Thang Pham, Huyen Thi Thanh Vu

**Affiliations:** 1 Department of Geriatrics, Hanoi Medical University, Hanoi 100000, Vietnam; nththu.bvlk@gmail.com (T.T.H.N.); thuhuonglk@hmu.edu.vn (H.T.T.N.); xuanthanh1901vlk@gmail.com (T.X.N.); ngoctamyhn@gmail.com (T.N.N.); nguyenlananh.bvlk@gmail.com (A.L.N.); phamthang@hmu.edu.vn (T.P.); vuthanhhuyen11@hmu.edu.vn (H.T.T.V.); 2 Scientific Research Department, National Geriatric Hospital, Hanoi 100000, Vietnam; 3 Institute for Global Health Innovations, Duy Tan University, Da Nang 550000, Vietnam; nguyenthuyentrang46@duytan.edu.vn (T.H.T.N.); vugialinh@duytan.edu.vn (L.G.V.); 4 Faculty of Medicine, Duy Tan University, Da Nang 550000, Vietnam; 5 Institute of Health Economics and Technology, Hanoi 100000, Vietnam; dothihoa.iheat@gmail.com (H.T.D.); dplinh.iheat@gmail.com (L.P.D.); 6 Center of Excellence in Evidence-Based Medicine, Nguyen Tat Thanh University, Ho Chi Minh City 700000, Vietnam; giang.coentt@gmail.com; 7 Dinh Tien Hoang Institute of Medicine, Hanoi 100000, Vietnam; huong.nguyen@dthim.org.vn; 8 Physiology Department, Hanoi Medical University, Hanoi 100000, Vietnam

**Keywords:** pain, older people, depression, chronic pain, Vietnam

## Abstract

The interaction of chronic pain and depression among older people has been studied for many years. This study aimed to investigate the frequency of chronic pain and depression among older patients and correlated factors. A cross-sectional study was conducted in 921 older patients at the National Geriatric Hospital from November 2019 to March 2020. We used the Charlson Comorbidity Index (CCI) to assess the comorbid condition, a numerical rating scale (NRS) to examine pain severity, and Geriatric Depression Scale—15 items (GDS-15) to measure depression among participants. A chi-square test and Tobit regression were used to analyze the relationships. A total of 921 older patients participated in the study. The proportion of depression accounted for 55.8%. The mean Charlson score and number of diseases were 1.2 and 4.7, respectively. A positive correlation was found between comorbidity and chronic pain and depression. Moreover, socio-demographic variables such as occupation, education, and income were associated with pain and depressive symptoms. This study highlights the issue of mental health in older people with chronic pain. The results indicate the necessity of frequent depression screening, pain management, and social activity programs for older people to enhance their health.

## 1. Introduction

The Vietnamese population is growing old rapidly. The proportion of older people accounted for 8.7% of the total population in 2009, which increased to 11.8% in 2019 [1]. This rapid transition might become a challenge for the healthcare system in order to ensure the health of the older population and improve health-related quality of life, including physical and mental health. Within those issues, chronic pain and depression are common physical and mental health problems in older people, which need proper prevention programs.

Older people with chronic pain also have a higher chance of functional disability compared to those younger [2]. Thus, these conditions have significant health and economic costs in some countries, where they have spent nearly 90% of the total health care cost for older adults [3]. Many studies have shown that mental health problems, such as anxiety, depression, and anger, are typically related to chronic pain [4]. Thus, the mental health problem in older people receives inadequate support from the government [5].

Mental health among older people also raises concerns from health care experts. Previous studies have shown that the prevalence of depression increases considerably among those who have chronic physical illnesses, including chronic pain [6,7,8]. Additionally, the percentage of older people in distress—physically, psychologically, or both—were high, accounting for 66% [9]. Many related factors might affect depression conditions, such as level of activity and demographic characteristics including age, income, gender, or behavior [10]. A previous study mentioned that nearly 70% of older people were at risk of mental health from mild to severe levels [11]. Depression in people with chronic pain frequently goes undiagnosed and untreated [6,11]. This may be due to the misunderstanding of depression among older people. Many people consider that depression is a part of the aging process [6]. On the other hand, depression does not have specific markers and objectives for diagnosis; thus, chronic pain and depression should be explored in research.

There have been few studies that have investigated depression and the associated factors related to depression conditions among older people in Vietnam. Therefore, this study aimed to describe the current situation of chronic pain and depression among older patients and the relationship between comorbidities, daily activities, chronic pain, and depression among this population.

## 2. Materials and Methods

### 2.1. Study Design

We conducted a cross-sectional study at the National Geriatric Hospital in Hanoi, Vietnam, from November 2019 to March 2020. We included the patients aged 60 years old and over with chronic pain at the National Geriatric Hospital. Eligible participants were invited to the private room and were introduced to this research. Registered nurses and nursing students provided information that made them fully understand the objectives, benefits, and shortcoming of the study. Those unable to communicate or who refused to participate in the interview during the recruitment process were excluded. There were a total of 921 participants in this study.

### 2.2. Measure and Instruments

#### 2.2.1. Socio-Economic Characteristics

Gender, age, educational level, occupation, marital status, living status, living area, health insurance, and monthly individual income were compiled from self-reports. We also asked participants about their behaviors related to smoking and alcohol consumption. Smoking status was characterized by current smoker, former smoker, and non-smoker. Alcohol use was self-reported and classified as drinking alcohol and no alcohol.

#### 2.2.2. Comorbidity

To examine the comorbidity of older patients, we used the Charlson Comorbidity Index (CCI), which categorized the comorbidity of patients from the hospital’s data [12]. To measure the participant’s comorbidity, we asked them to report their current pathological as well as acute health status, including myocardial infarction, congestive heart failure, peripheral vascular disease, dementia, chronic lung disease, cerebrovascular disease, and peptic ulcer.

#### 2.2.3. Pain Characteristics

Chronic pain has been defined as pain that persists or re-occurs, lasting longer than 3 months [13].

To assess pain intensity, we used a numerical rating scale (NRS), which consists of 10 points from 0 (no pain) to 10 (worst possible pain) [14]. Patients were asked to choose a score from 0 to 10 to describe their pain intensity [15,16].

Pain location (head–face–neck, shoulder–arm–elbow–hand, stomach–abdomen, back–hip, knee–foot–leg, or other), number location of pain and satisfaction level with current pain medications (very unsatisfied, partly unsatisfied, satisfied, partly satisfied, and very satisfied) were included.

#### 2.2.4. Physical Activity

To assess physical activity, we used the International Physical Activity Questionnaire (IPAQ), which included moderate and vigorous activity and walking in the past 7 days. Physical activity levels were categorized into low, moderate, and high levels based on the total score [17].

#### 2.2.5. Depression

The 15-item Geriatric Depression Scale, Vietnamese version, was used to screen the depression condition in the older patients. The total scores range from 0 to 15, in which scores of 0–5 are considered normal and 6–15 indicate having depressive symptom [5,18].

### 2.3. Statistical Analysis

We described qualitative variables, socio-demographic, and the geriatric depression score among samples by using mean, standard deviation, number, and percentage. Statistically significant data were indicated at the 95% confidence level (*p* < 0.05). Comparisons between participants with depressive symptom and non-depressive symptom were assessed by using Chi-squared test for categorical variables and Student’s t-tests for continuous variables. Tobit regression was used to examine factors associated with depression condition and pain intensity; the coefficient and 95% CI (confident intervals) were also calculated. Stepwise with *p*-values ≤ 0.2 for the Wald test was used to include variables in the model. The STATA software version 14.0 (Stata Corp. L.P., College Station, TX, USA) was adopted to perform all statistical analyses.

## 3. Results

Among 921 older patients, the mean age was 72.6 years (SD = 9.2). The prevalence of older patients with depressive symptoms was 56.5%. The mean age of older patients with depressive symptoms was 73.5 (9.5) years; two thirds were female, and 84.9% lived with their family. The proportion of people with health insurance was 95.1%, and approximately two thirds had a monthly income of ≤3,000,000 VND (approximately 129.47 USD) (Table 1).

The pain characteristics of participants are displayed in Table 2. The proportion of pain in head–face–neck was highest in the group with depressive symptoms. Statically significant differences in depressive symptoms were found for pain located in the head–face–neck and knee–foot–leg areas, using pain medication and Charlson score. The proportion of patients using pain medication in the depressive symptom group was 56.9%, higher than those without depressive symptom (Table 2).

Table 3 presents the health-related behavior among older patients. Of the older patients in the depressive symptom group, 25.3% reported lifetime smoking, and 6.9% of those were current smokers. There were 119 (26.9%) patients with a high physical activity level and 213 (48.2%) patients with a moderate level, defined using the IPAQ questionnaire. The relationships between depressive symptoms and regular exercise and physical activity were statistically significant.

Table 4 presents odds ratios (ORs) and 95% CI from the multiple logistic regression analysis of relationships between participants with depressive symptoms and related factors and a Tobit regression analysis of relationships between the numeric rating scale of pain and related factors. There was a statistically significant association between monthly income, pain location in head–face–neck and shoulder–arm–elbow–hand, satisfaction level with current pain medications, and numeric rating scale in pain and depressive symptoms.

## 4. Discussion

Our study highlights a high proportion of patients with depression among study samples, at 56.5%. Otherwise, the most common pain locations of the participants were head–face–neck and knee–foot–leg. Participants with moderate and high physical activity levels had better mental health conditions. These findings suggested the improvement of primary care for older people at a community level.

It is clear that older people become more sensitive to chronic illnesses, including chronic pain, while also being at higher risk of falling, frailty, and difficulty performing daily activities [2]. In this analyzed sample, the prevalence of depressive symptoms in women was higher than in men; this result is in line with a study in the United Kingdom, which also showed that women were more likely to indicate depressive symptoms than were men [19]. Undoubtedly, chronic pain is one of the obstacles to daily life in the elderly [2,20,21]. In the health context of Vietnam, there is a lack of medical facilities at the community level, and rehabilitation for older adults has not focused appropriately. These results also indicate that the early detection of depressive symptoms in patients with chronic pain is a great and important requirement for comprehensive geriatric assessment in hospitals. Age significantly interacts with pain severity, especially people over 70 years old. This is consistent with a study from China [22]. Therefore, pain management should be modified and designed for different ages and genders.

In addition to the pain issues, our research results also showed a correlation between comorbidity and depression in older patients. The results indicated that approximately 60% of the participants had a depressive symptom. This number was higher than a study conducted in a rural area in Vietnam, with 26.4% at risk for depression [5]. It is explained that older patients with chronic pain tend to develop depressive symptoms more often than older people in rural areas with non-chronic pain. The proportion of depressive symptoms in our sample is similar to that in the study from Lauren R [22]. Considering additional probably related factors of depression among the elderly, we found some positive associated factors mentioned above, such as pain location [19,21] and comorbidity [9]. Multiple pain sites and a history of depression are factors that predict low outcomes. To tackle this problem, public health programs need to be more specific, in particular to support counseling and relieve depression in older people. Health workers need to be trained in health counseling, especially mental health. On the other hand, a lower monthly income was found to be associated with the likelihood of having a mental health problem, which is somewhat in line with the study of Mojtabai R [23,24]. It can be assumed that older people who had a monthly income might not have an economic burden or can afford their daily needs. In contrast, living in a rural area or earning less money can be an obstacle for older people in daily life, especially when they live alone [5]. Thus, it is important to establish chronic pain management programs for poor people in rural and mountainous areas.

As in our hypothesis, physical activity plays an essential role in the relationship between chronic pain and depression. In the current study, after adjusting for possible confounding factors, the elderly who had moderate and high physical daily activity were less likely to suffer a high intensity of pain. Moderate and high physical activity levels were associated with non-depressive symptoms in univariate analysis but were not statistically associated in the Tobit regression model. However, the result showed that moderate and high physical activity levels tend to decrease the pain severity. This result was similar with the finding in China [22]. It can be explained that doing physical activities might help people to strengthen the stretch of their low back, head–neck location, or muscles. Moreover, the effectiveness of physical therapy in chronic pain management was investigated [25,26]. Additionally, when older people doing physical activities enjoy doing exercise, riding a bike, playing any kind of sport, they would prefer to do it in a group of people, which may help them to open their social network and reduce the chance of being alone and at risk of depression. Therefore, those activities might decrease the symptom of depression [11,27,28]. It is suggested that developing occupational therapy and physical therapy training in Vietnam is critical. On the other hand, engaging social networks should be indicated in hospital, because, in Vietnam, social workers are still lacking in the healthcare system. There are several implications in our study. First, our results reported the current prevalence risk for depression among older people with chronic pain, which should not be misjudged or considered a part of aging. It can be seen from a clinical perspective that we should not underestimate physical examination. Physical activities in older people not only correlate with pain but are also associated with depression, including people with low physical pain. Therefore, we need to work towards homecare service and develop policies to promote activities for older adults of both rural and urban areas, which might help older adults receive healthcare at home. Our research has some limitations. The cross-sectional study might mean it is difficult to conclude the causal relationship between socio-demographic characteristics and other variables and outcomes, which demand further study.

## 5. Conclusions

This study highlights the issue of mental health in older people with chronic pain. The results indicated the necessity of frequent depression screening, pain management, physical activity programs, and social activity programs for older people to enhance their health. Future longitudinal studies should explore the relationship between socio-demographic characteristic and physical activity with chronic pain and pain-related outcomes.

## Figures and Tables

**Table 1:** Demographic characteristics of respondents by depressive symptoms.

	Depressive Symptoms 520 (56.5%)		Non-Depressive Symptoms 401 (43.5%)		All		*p*-Value
	n	%	n	%	n	%	
Gender							
Male	173	33.3	134	33.4	307	33.3	0.96
Female	347	66.7	267	66.6	614	66.7	
Age							
≤70 years old	226	43.5	197	49.1	423	45.9	0.09
>70 years old	294	56.5	204	50.9	498	54.1	
Occupation							
Retired	437	84.0	325	81.0	762	82.7	0.23
Working	83	16.0	76	19.0	159	17.3	
Marital status (missing 5)							
Single	192	37.3	98	24.6	290	31.7	<0.01
Married	323	62.7	301	75.4	624	68.3	
Living status							
Living with family	425	81.7	357	89.0	782	84.9	<0.01
Alone	95	18.3	44	11.0	139	15.1	
Self-monthly income (missing 3)							
≤3,000,000 VND	355	68.7	245	61.3	600	65.4	0.02
>3,000,000 VND	162	31.3	155	38.8	317	34.6	
Health insurance							
No	494	95.0	382	95.3	876	95.1	0.86
Yes	26	5.0	19	4.7	45	4.9	
Living area							
Urban	291	56.0	221	55.1	512	55.6	0.80
Rural	229	44.0	180	44.9	409	44.4	
Educational level (missing 1)							
<High school	125	24.1	86	21.4	211	22.9	0.15
High school	137	26.4	115	28.7	252	27.4	
>High school	166	32.0	147	36.7	313	34.0	
Others	91	17.5	53	13.2	144	15.7	
	Mean	SD	Mean	SD	Mean	SD	*p*-value
Age	73.5	9.5	71.4	8.6	72.6	9.2	<0.01

**Table 2:** Pain characteristics in participants (n = 921).

	Depressive Symptoms 520 (56.5%)		Non-Depressive Symptoms 401 (43.5%)		Total		*p*-Value
	n	%	n	%	n	%	
Pain location							
Head–Face–Neck	227	43.7	101	25.2	328	35.6	<0.01
Shoulder–Arm–Elbow–Hand	109	21.0	86	21.4	195	21.2	0.86
Stomach–Abdomen	61	11.7	55	13.7	116	12.6	0.37
Back–Hip	102	19.6	81	20.2	183	19.9	0.83
Knee–Foot–Leg	158	30.4	161	40.1	319	34.6	<0.01
Other	135	26.0	71	17.7	206	22.4	<0.01
Number location of pain							
0	58	11.2	47	11.7	105	11.4	0.88
1	308	59.2	241	60.1	549	59.6	
≥2	154	29.66	113	28.2	267	29.0	
Satisfaction level with current pain medications							
Very satisfied	5	1.8	2	1.2	1.59	1.6	0.1
Partly satisfied	20	7.2	20	12.2	40	9.1	
Satisfied	201	72.6	124	75.6	325	73.7	
Partly unsatisfied	46	16.6	17	10.4	63	14.3	
Very unsatisfied	5	1.8	1	0.6	6	1.4	
	Mean	SD	Mean	SD	Mean	SD	*p*-value
Charlson score	1.6	1.4	1.3	1.5	1.5	1.5	<0.01
NRS score	4.6	1.8	4.5	1.5	4.6	1.7	0.66

**Table 3:** Health-related behavior among older people.

Characteristics	Depressive Symptoms		Non-Depressive Symptoms		Total		*p*-Value
	n	%	n	%	n	%	
Current smoker	26	6.9	16	4.9	42	6.0	0.27
Former smoker	92	25.3	69	21.7	161	23.6	0.26
Drinking alcohol	61	76.3	39	75.0	100	75.8	0.87
Regular exercise	153	29.9	166	41.9	319	35.2	<0.01
Physical Activity (IPAQ)							
Low	110	24.9	35	9.8	145	18.2	<0.01
Moderate	213	48.2	198	55.6	411	51.5	
High	119	26.9	123	34.6	242	30.3	

**Table 4:** Factors associated with depression among the participants.

	NRS		Depressive Symptoms	
	Coef	95% CI	Coef	95% CI
Socio-economic characteristic				
Gender (Male = ref)	−0.32 *	(−0.64–0.01)		
Occupation (retired = ref)				
Working	0.40 *	(−0.06–0.86)		
Monthly income (≤3,000,000 VND = ref)				
>3,000,000 VND			−0.57 **	(−1.12–−0.03)
Age (≤70 years old = ref)				
>70 years old	0.36 **	(0.01–0.70)		
Physical activity				
IPAQ (Low = ref)				
Moderate	−0.46 **	(−0.83–−0.09)		
High	−1.87 ***	(−2.36–−1.38)		
Pain and Comorbid				
Charlson comorbidity	0.15 ***	(0.05–0.26)		
Pain location (No = ref)				
Head–Face–Neck			0.83	(0.29–1.37)
Shoulder–Arm–Elbow–Hand			0.66	(0.03–1.29)
Satisfaction level with current pain medications (Very satisfied = ref)				
Partly satisfied	0.42	(−0.08–0.92)	−1.76 ***	(−2.77–−0.74)
Partly unsatisfied			−0.68 *	(−1.37–0.01)
NRS			0.23	(0.08–0.38)

*: *p* < 0.05; **: *p* < 0.01; ***: *p* < 0.001.

## Data Availability

The datasets of this study are available from the corresponding author on reasonable request.
